# Supplementary material for: Encouraging perspective taking: Using narrative writing to induce empathy for others engaging in negative health behaviors
Source: PLoS One. 2019 Oct 15;14(10):e0224046. doi: 10.1371/journal.pone.0224046 (PMC6793876; doi:10.1371/journal.pone.0224046)
Supplement: S1 Appendix — (PDF) [file pone.0224046.s001.pdf]

ParticipantNumber

Participant Number

Cover Letter

The purpose of this study is to examine situational, and personality differences in how people write narrative essays. To test this, we will first ask you to complete several questions related to a hypothetical situation. Then we will ask you to write a narrative essay. After writing, we will ask you to complete another set of questions.

If you choose to participate, you will complete a study that will take about 60 minutes. Your responses will be confidential; we will not collect any identifiable information within the survey itself.

Participation in this study is voluntary. If you choose to participate, you will receive two half-hour research credits for completing the survey. There will be alternate assignments if you do not wish to participate in this study.

If you choose to participate in this study, please click on the arrow in the lower right hand corner to begin.

**To get credit for this survey, you must reach the very last page and submit your responses. The page will say: "We thank you for your time spent taking this survey. Your response has been recorded."

These page timer metrics will not be displayed to the recipient.

First Click: *0 seconds*

Last Click: *0 seconds*

#QuestionText, TimingPageSubmit#: *0 seconds*

#QuestionText, TimingClickCount#: *0 clicks*

Pre-intervention Questions

To begin, please imagine the following scene:

You are leaving a grocery store. You notice that in the parking lot there is a pregnant woman smoking a cigarette.

These page timer metrics will not be displayed to the recipient.

First Click: *0 seconds*

Last Click: *0 seconds*

#QuestionText, TimingPageSubmit#: *0 seconds*

#QuestionText, TimingClickCount#: *0 clicks*

Please rate your imagined emotional state after viewing this pregnant woman smoking.

I would feel the following emotions toward this woman:

	Not at all 0	A great deal 100
Sadness		
Excitement		
Anger		
Pity		
Disgust		
Happiness		
Hopelessness		
Surprise		
Disappointment		
Concern		

Please rate your agreement with the following statements.

After viewing this scene, I would think that:

This woman is a bad mother.

	Strongly Disagree 0	Strongly Agree 100

This woman is selfish.

Strongly Disagree Strongly Agree

0 100

This woman is doing the best she can.

Strongly Disagree Strongly Agree

0 100

This woman does not have her future child's best interest at heart.

Strongly Disagree Strongly Agree

0 100

Please rate your agreement with the statements below.

When you were considering how you feel about this person and her behavior, to what extent did you do the following?

I put myself “in her shoes”.

Strongly Disagree Strongly Agree

0 100

I felt very sorry for her when I was thinking about her problems.

Strongly Disagree Strongly Agree

0 100

I tried to take her side of the problem.

Strongly Disagree Strongly Agree

0 100

I felt kind of protective towards her.

Strongly Disagree Strongly Agree

0 100

I imagined what it was like to be her.

Strongly Disagree Strongly Agree

0 100

Her misfortunes disturbed me a great deal.

Strongly Disagree Strongly Agree

0 100

I tried to look at her side of the situation in addition to my own.

Strongly Disagree Strongly Agree

0 100

I felt pity for her when I was thinking about her experience.

Strongly Disagree Strongly Agree

0 100

I found it difficult to see things from her point of view.

Strongly Disagree Strongly Agree

0 100

Before criticizing her, I tried to imagine how I would feel if I were in her place.

Strongly Disagree Strongly Agree

0 100

Based on my feelings while considering this scene, I would describe myself as a pretty soft-hearted person.

Strongly Disagree Strongly Agree

0 100

I was sure I was right about her, so I don't waste much time considering her side of the situation.

Strongly Disagree Strongly Agree

0 100

I didn’t spend lots of time trying to get her point of view.

Strongly Disagree Strongly Agree

0 100

I tried to imagine how things look from her perspective.

Strongly Disagree Strongly Agree

0 100

To what extent is this person to blame for her action of smoking cigarettes while pregnant?

Not at all A great deal

0 100

To what extent are external factors, such as life circumstances, responsible for this person smoking cigarettes while pregnant?

Not at all A great deal

0 100

To what extent does this person have freedom to make better choices?

Not at all A great deal

0 100

Please indicate the extent of your agreement with the following statement:

I can never imagine a situation where I would smoke cigarettes while pregnant.

Strongly Disagree Strongly Agree

0 100

First Scene_Control Condition

Get your thinking cap on! It's time to write your narrative!

First scene prompt:

Please describe the room you are currently sitting in. Please write about different features of the room, including what you can see from your desk, details about the space and the objects in it. Where are you? What is around you? Remember to use sensory details such as sounds, smells, air temperature, etc.

*Please write as much as you can for the next **ten minutes**.*

These page timer metrics will not be displayed to the recipient.

First Click: *0 seconds*

Last Click: *0 seconds*

#QuestionText, TimingPageSubmit#: *0 seconds*

#QuestionText, TimingClickCount#: *0 clicks*

Second Scene_Control

Second scene:

Please describe what you first saw as you entered the building and walked to this room. Please describe the features of the building and the room in as much detail as possible.

*Please write as much as you can for the next **five minutes**.*

Narrative Intervention_Experimental Condition

Something that is often difficult for people to understand is why someone might smoke cigarettes while pregnant.

Earlier, we asked you to imagine that you are leaving a grocery store. You notice that in the parking lot there is a pregnant woman smoking a cigarette. Please think about this woman.

Take a mental snapshot of that person and answer the following questions about your mental snapshot.

Her age is:

- Younger than 16
- Between 16-21
- Between 21-29
- Over 30

Her race is:

- White/Caucasian
- Black/African American
- Asian/Pacific Islander
- American Indian/Alaska Native

- Hispanic/Latina
- Other

If other, please specify:

She lives in a place that can be described as:

- Urban
- Rural
- Medium-sized city

Her economic status is best described as:

- Very poor
- Working poor
- Middle-class
- Upper middle class
- Wealthy

The highest level of education she received is:

- Did not finish high school
- Finished high school
- Some college
- Finished college
- Graduate or professional school

Exp_CharacterDevelopment

Now, you will be asked to do some creative writing about this character you've imagined. Don't worry if you've never written fiction before, you will be given prompts to help you. It's also okay if you use some details from real life to create your fictional character and scenes. The goal is to try to imagine them, and write about them, as vividly as you can.

First, think of a name for this person:

Please enter her name here:

Now, use your imagination to answer the following questions about your character.

What was her favorite present on her 10th birthday?

When she wakes up in the morning, the first thing she thinks of is….

Her biggest fear is that….

When she walks into a room full of strangers, the first thing she thinks is….

The thing that makes her feel most hopeful is…

No one might ever guess that she can…

The thing/person that she loves most is..

If she had to say what she needed most she would say…

These page timer metrics will not be displayed to the recipient.

First Click: *0 seconds*

Last Click: *0 seconds*

Page Submit: *0 seconds*

Click Count: *0 clicks*

Exp_SceneExamples

Next, you will be given some space to write a scene that features your character at a particular moment in her life. A scene is a piece of writing that describes something happening the way that a movie camera might film it—in real time. The person or people in the scene are seeing things, doing things, feeling and thinking things. They are even hearing and smelling things.

If you would like **examples of scenes**, click below. If not, click the arrow button to proceed.

◯ (Examples of scenes will appear on the next page)

These page timer metrics will not be displayed to the recipient.

First Click: *0 seconds*

Last Click: *0 seconds*

Page Submit: *0 seconds*

Click Count: *0 clicks*

Exp_Scene Examples2

Scene Examples:

“We dumped our tent and our sleeping bags near a basalt grotto with a spring bubbling from it and Gordon said, “Let’s go, troops,” holding his rifle before his chest diagonally, as a soldier would. He dressed as a soldier would too, wearing his father’s over-large cammies rather than the mandatory blaze-orange gear. Fifty feet apart, we worked our way downhill through the forest, through a huckleberry thicket, through a clear-cut crowded with stumps, taking care not to make much noise or slip on the pine needles carpeting the ground. A chipmunk worrying at a pinecone screeched its astonishment when a peregrine falcon swooped down and seized it, carrying it off between the trees to some secret place. Its wings made no sound, and neither did the blaze-orange-clad hunter when he appeared in a clearing several hundred yards below us. Gordon made some sort of SWAT-team gesture—meant, I think, to say, stay low—and I made my way carefully toward him. From behind a boulder we peered through our scopes, tracking the hunter, who looked, in his vest and ear-flapped hat, like a monstrous pumpkin.” **Short story, *Refresh, Refresh* (published 2006) by Benjamin Percy**

“She turned and bumped against a chair or something, hurting her leg, but she ran into the back room and picked up the telephone. Something roared in her ear, a tiny roaring, and she was so sick with fear that she could do nothing but listen to it—the telephone was clammy and very heavy and her fingers groped down to the dial but were too weak to touch it. She began to scream into the phone, into the roaring. She cried out, she cried for her mother, she felt her breath start jerking back and forth in her lungs as if it were something Arnold Friend was stabbing her with again and again with no tenderness. A noisy sorrowful wailing rose all about her and she was locked inside it the way she was locked inside this house." **Novel, *Where Are You Going, Where Have You Been* (published 1966) by Joyce Carol Oates**

These page timer metrics will not be displayed to the recipient.

First Click: *0 seconds*

Last Click: *0 seconds*

Page Submit: *0 seconds*

Click Count: *0 clicks*

Exp_Writing Tips

If you would like **tips about writing scenes**, click below. If not, click the arrow button to proceed.

◯ (Tips about writing scenes will appear on the next page)

Exp_Writing Tips2

Writing Tips:

- Don't try to pack a lot of background information into the scene. Just describe what is happening now.
- Remember to use sensory detail—describe what your character sees, smells and hears.
- Describe what is going on around them, or objects in the room.
- What is your character thinking and feeling while the things you describe are happening?

These page timer metrics will not be displayed to the recipient.
First Click: *0 seconds*
Last Click: *0 seconds*
Page Submit: *0 seconds*
Click Count: *0 clicks*

Exp_CharacterReminder

As a reminder, your character has the following characteristics:

Her age is: Younger than 16

Her age is: Between 16-21

Her age is: Between 21-29

Her age is: Over 30

Her race is: White/Caucasian

Her race is: Black/African American

Her race is: Asian/Pacific Islander

Her race is: American Indian/Alaska Native

Her race is: Hispanic/Latina

Her race is: Other

She lives in a place that can be described as: Urban

She lives in a place that can be described as: Rural

She lives in a place that can be described as: Medium-sized city

Her economic status is best described as: Very poor

Her economic status is best described as: Working poor

Her economic status is best described as: Middle-class

Her economic status is best described as: Upper middle class

Her economic status is best described as: Wealthy

The highest level of education she received is: Did not finish high school

The highest level of education she received is: Finished high school

The highest level of education she received is: Some college

The highest level of education she received is: Finished college

The highest level of education she received is: Graduate or professional school

First Scene_Experimental Condition

Get your thinking cap on! It's time to write your narrative!

First scene prompt:

Please describe a scene in which your character is on her way to work. Where is she? Is she alone? What is around her? Remember to use sensory details such as sounds, smells, air temperature, etc. If you want, you can include dialogue.

Please write as much as you can for the next ten minutes.

While writing about this character, please adhere to one rule: you must assume this person is at least as smart as you are.

By "smart", we do not mean "educated". Your character may not be very educated (e.g., did not complete high school) but she still must be as intelligent as you are.

These page timer metrics will not be displayed to the recipient.

First Click: *0 seconds*

Last Click: *0 seconds*

Page Submit: *0 seconds*

Click Count: *0 clicks*

Second Scene_Experimental Condition

Second scene:

Please describe a scene in which your character is attempting to do something difficult with another person (Fix a car? Cook a meal? Bind a wound?) and something goes wrong.

Again, while writing about this character, please adhere to one rule: you must assume this person is at least as smart as you are.

By "smart", we do not mean "educated". Your character may not be very educated (e.g., did not complete high school) but she still must be as intelligent as you are.

Please write as much as you can for the next five minutes.

These page timer metrics will not be displayed to the recipient.
First Click: *0 seconds*
Last Click: *0 seconds*
Page Submit: *0 seconds*
Click Count: *0 clicks*

Post-Intervention Questions1

Please answer the following questions.

To what extent do you feel like you know why some women smoke cigarettes while pregnant?

Not at all Very much

0 100

Why do you think some women smoke cigarettes while pregnant?

Overall, do you feel badly for women who smoke cigarettes while pregnant?

Not at all 1	Slightly 2	Moderately 3	Very 4	Extremely 5
◯	◯	◯	◯	◯

Now we are going to ask you a second set of questions.

Again, imagine that you are leaving a grocery store. You notice that in the parking lot there is a pregnant woman smoking a cigarette.

Please rate your imagined emotional state after viewing this pregnant woman smoking.

I would feel the following emotions toward this woman:

	Not at all 0	A great deal 100
Sadness		
Excitement		
Anger		
Pity		
Disgust		
Happiness		
Hopelessness		
Surprise		
Disappointment		
Concern		

Please rate your agreement with the following statements.

After viewing this scene, I would think that:

This woman is a bad mother.

Strongly Disagree Strongly Agree

0 100

This woman is selfish.

Strongly Disagree Strongly Agree

0 100

This woman is doing the best she can.

Strongly Disagree Strongly Agree

0 100

This woman does not have her future child's best interest at heart.

Strongly Disagree Strongly Agree

0 100

Please rate your agreement with the statements below.

When you were considering how you feel about this person and her behavior, to what extent did you do the following?

I put myself “in her shoes”.

Strongly Disagree Strongly Agree

0 100

I felt very sorry for her when I was thinking about her problems.

Strongly Disagree Strongly Agree

0 100

I tried to take her side of the problem.

Strongly Disagree Strongly Agree

0 100

I felt kind of protective towards her.

Strongly Disagree Strongly Agree

0 100

I imagined what it was like to be her.

Strongly Disagree Strongly Agree

0 100

Her misfortunes disturbed me a great deal.

Strongly Disagree Strongly Agree

0 100

I tried to look at her side of the situation in addition to my own.

Strongly Disagree Strongly Agree

0 100

I felt pity for her when I was thinking about her experience.

Strongly Disagree Strongly Agree

0 100

I found it difficult to see things from her point of view.

Strongly Disagree Strongly Agree

0 100

Before criticizing her, I tried to imagine how I would feel if I were in her place.

Strongly Disagree Strongly Agree

0 100

Based on my feelings while considering this scene, I would describe myself as a pretty soft-hearted person.

Strongly Disagree Strongly Agree

0 100

I was sure I was right about her, so I don't waste much time considering her side of the situation.

Strongly Disagree Strongly Agree

0 100

I didn’t spend lots of time trying to get her point of view.

Strongly Disagree Strongly Agree

0 100

I tried to imagine how things look from her perspective.

Strongly Disagree Strongly Agree

0 100

To what extent is this person to blame for her action of smoking cigarettes while pregnant?

Not at all A great deal

0 100

To what extent are external factors, such as life circumstances, responsible for this person smoking cigarettes while pregnant?

Not at all A great deal

0 100

To what extent does this person have freedom to make better choices?

Not at all A great deal

0 100

Please indicate the extent of your agreement with the following statement:

I can never imagine a situation where I would smoke cigarettes while pregnant.

Strongly Disagree	Strongly Agree
0	100

In your opinion, what is the best way to reduce the incidence of women smoking cigarettes while pregnant?

Please select your ***top three*** recommendations for ways to reduce the incidence of women smoking cigarettes while pregnant: **(pick only 3)**

- ☐ mandatory jail for women who smoke while pregnant
- ☐ monetary fine for women who smoke while pregnant
- ☐ educating all women on the danger of smoking while pregnant
- ☐ greater social support for pregnant mothers
- ☐ greater social services for low-income women (help with employment, child care, housing)
- ☐ free health care for men and women
- ☐ comprehensive smokefree policies in local communities
- ☐ comprehensive statewide smoke free policy
- ☐ increase the state cigarette tax
- ☐ free smoking-cessation programs for men and women

Follow-Up Questions

Now that you have completed this exercise, we would like to ask you some follow-up questions. Please complete the following items:

How difficult was it to write your narratives?

Very Difficult	Difficult	Somewhat Difficult	Neutral	Somewhat Easy	Easy	Very Easy
○	○	○	○	○	○	○

What are your thoughts about the exercise you just completed?

Did you understand the purposes of the study you just completed?

- ○ Yes
- ○ No

Please explain:

Demographics

Lastly, please answer the following demographic questions.

You are free to skip any of these questions

Do you smoke cigarettes?

- Yes
- No

At any point in your childhood, did one or both of your parents smoke cigarettes?

- Yes
- No
- I don't know/not applicable

At any point in your childhood, did your mother smoke cigarettes?

- Yes
- No
- I don't know/not applicable

What is your gender?

- Male
- Female

What is your age? (in years)

How would you best characterize your race/ethnicity? (check all that apply)

- Caucasian/White
- African-American/Black
- Asian/Pacific Islander
- American Indian/Alaska Native
- Hispanic/Latino(a)
- Other

What is your year in school?

- Freshman
- Sophomore
- Junior
- Senior

Debriefing

Debriefing

Thank you for participating in this study! I want to take a few additional minutes to tell you a little more about this research and why we are doing it.

Preliminary work has suggested that writing narratives about a person can increase empathy toward that person and a greater consideration of external causes for that person's behavior (e.g., a woman who smokes while pregnant). Based on this preliminary research, narrative writing may be a particularly important intervention for medical professionals and policy makers who might develop better policy or treatment recommendations if they had a better understanding of how external forces can play a role in someone's behavior

To test our idea, we asked introductory psychology students to write a narrative. Some participants were randomly assigned to write about a woman who smokes cigarettes while pregnant – a behavior that is often difficult for people to understand, and a behavior that people traditionally attribute to the person's individual characteristics (as opposed to societal influences, like poverty, that might contribute to this unhealthy behavior). Some other participants were asked to write about they room they are currently sitting in. These participants were in our "control condition" which allows us to compare their responses to people who were asked to write a narrative about another personal

Hence, this study used a between-subjects pre-post design. By between-subjects, we mean that some participants were asked to write one kind of essay and others were asked to write a different kind of essay – specifically, some were asked to write about a woman who smokes while pregnant and others were asked to write about the room they are sitting in. To measure the effectiveness of our intervention (i.e., writing the narrative) we measured participants' feelings and attitudes before the intervention (pre) and then after the intervention (post). Thus, a "pre-post" design.

Participants were first asked several questions about their attitudes toward a mother who smokes cigarettes while pregnant. We also asked people to report their empathy toward the woman and their attitudes toward her behavior. They were then asked to write a narrative about either: a woman who smokes cigarettes while pregnant or the room they are sitting in. For those who were asked to write about a woman who smokes while pregnancy, we asked them to think of her as a whole person – not just a single aspect of her behavior like smoking while pregnant. We think this is important because writing about her life and experiences more broadly should allow people to see her more fully and better understand how her social context might play a role in her behavior. After writing the narrative, participants were again asked the same questions then completed before writing the narrative. We also asked participants for suggestions for ways to reduce the likelihood that women will smoke cigarettes while pregnant. To understand whether the intervention "worked", we will compare participants' responses before and after the intervention.

We hypothesize that writing a narrative about a person and the possible reasons for their unhealthy choices will cause people to be H1) more empathetic toward the person, H2) have a stronger understanding of external factors that influence people's behavior, and H3) create interventions to reduce the person's harmful behavior that have less to do with the individual and more to do with society. We reason that if this narrative writing intervention increases empathy and an understanding of how external forces influence behavior, people should be more likely to endorse policies that focus on changing environmental factors and institutions rather than ones that focus on fixing the individual.

We really appreciate your time in helping us with this research and are always happy to answer any questions that you might have about it. We think that learning more about the kinds of issues we investigated today can be used to improve the ways health-care professionals interact with their clients. We also think it can improve ways that policy makers (in government and health-care organizations) create interventions to promote health and well-being. We hope that your responses to today's study will help us

answer these questions.

If you have any additional questions about the study, you can reach me by email (focellae@uwosh.edu).
Thank you again for your participation.

-Dr. Elizabeth Focella
