# Supplementary material for: Encouraging perspective taking: Using narrative writing to induce empathy for others engaging in negative health behaviors
Source: PLoS One. 2019 Oct 15;14(10):e0224046. doi: 10.1371/journal.pone.0224046 (PMC6793876; doi:10.1371/journal.pone.0224046)
Supplement: Attachment — Submitted filename: Narrative Writing Manuscript Response to Reviewers.docx [file pone.0224046.s002.docx]

Paula Pérez-Sobrino, PhD

Academic Editor, *PLOS ONE*

Dear Dr. Pérez-Sobrino,

Thank you for the feedback on the revised version of our manuscript. We used the comments from the two reviewers to further refine the paper. In this letter, I will detail the changes that we have made to the manuscript in response to these comments. As requested, we have uploaded both a copy of the revised manuscript with the changes tracked and an unmarked copy.

**Editor Comments:**

Comment: Please ensure that your manuscript meets PLOS ONE’s style requirements.

Response: We updated our manuscript and file names accordingly.

Comment: Please provide additional details regarding participant consent. In the ethics statement in the Methods and online submission information, please ensure that you have specified (1) whether consent was informed and (2) what type you obtained (for instance, written or verbal, and if verbal, how it was documented and witnessed). If your study included minors, state whether you obtained consent from parents or guardians. If the need for consent was waived by the ethics committee, please include this information.

Response: Neither study included minor participants. This is clarified in the Participants section of both studies (see pages 10 and 16). We provided additional details about the consent process for both studies in the Materials and Procedure subsections (see pages 10-11 and page 16).

**Reviewer 1**:

Comment: Abstract: presents the central themes sufficiently but can be more clear when it is presented in a structure such as: goal, method, results, discussion, conclusion.

Response: It is our understanding that the PLOS One journal style requires unstructured abstracts, so we did not modify the abstract as suggested.

Comment: Introduction: describes important topics, however it is too much and too long. Many topics belong, in my opinion, to the discussion. For instance in a paragraph as “comparison with other research”. Furthermore dated references are used (1993, 1997).

Response: We streamlined the Introduction and reduced the text by approximately 1,000 words. However, we disagree that the date of publication should be a reason to exclude citations from the manuscript. While we cite a handful of classic references, the majority of the research cited in the manuscript are much more recent publications.

Comment: At the end of the discussion a description of a precise gap in research is lacking and the goal of the study has been described ambiguously.

Response: We revised the Introduction to include a precise description of the gap in the literature and described the goals of the two studies more explicitly (pages 6-7)

Comment: After the general introduction a new introduction starts of study 1, that is confusing.

Response: We removed the separate introductions to the two studies.

Comment: Method: is a very long paragraph. In my opinion it is useful to summarize. Moreover sometimes the method is mixed up with items from the results paragraph.

Response: We improved the efficiency of our Method section by removing material that was redundant with the Introduction. However, we prefer to err on the side of greater transparency and retain many of the methodological details. We moved the details about our analytical methods from the Results section to the Method section.

Comment: Results: after the results the description of the second study starts and after this description a discussion starts. And the discussion lacks a clear arrangement (summary of the study, strengths and limitations, comparison with existing literature).

Response: We added a section on the Strengths and Limitations of the research and included headers to emphasize the structure of the Discussion section (pages 22-25)

**Reviewer 2**:

Comment: Overall Impression: This study has value for interventions that may help mitigate bias, promote perspective-taking and empathy, and foster considerations for the impact of social determinants of health on patient decision-making and health outcomes.

Response: Thank you!

Comment: It may be slightly awkward to start the first sentence of the abstract with “People,”. Perhaps something like, “It is very common for people, including health professionals….” OR “Societal expectations of self-care and responsible actions toward others may produce bias against those who engage in perceived self-harming behavior. This is especially true for health professionals, who have dedicated themselves to helping reduce the burden of illness and suffering.”

Response: We appreciate the suggestion and have modified the first two sentences of the Abstract to include your suggested wording.

Comment: In the last sentence of the abstract, the definition of the adjective “dubious” with regard to behavior does not seem to align with the authors’ intended use. Perhaps “irresponsible,” “self-harming” or “adverse” would be clearer to readers?
Response: We replaced ‘dubious’ with ‘irresponsible’.

Comment: Typo, top of page 16, “. There were no prepost differences in reported hopelessness, sadness, excitement, happiness, or concern, p > ,05.” – comma should be replaced with a period to read “p> 0.05.”

Response: We corrected the punctuation.

Comment: Page 20, for the sentence, “There were no significant time by condition interactions observed for sadness, excitement, happiness, concern, and hopelessness, p > .005.” – did you mean to say “p> 0.05.” rather than 0.005? My understanding is that you were stating that the results in these domains were not significant (eg, p not equal to or less than 0.05), correct?

Response: Because we had no *a priori* predictions regarding emotions, we employed a Bonferroni correction for multiple comparisons, which resulted in a critical alpha value of .005. However, we reminded the reader about the use of the Bonferroni procedure in that paragraph to eliminate confusion.

Comment: Statements in the Results section, such as, “This is unsurprising given that one of the two writing prompts asked participants to specifically write about their character engaging in a difficult situation.” would likely be better placed into the Discussion Section. Results should be stated as such, with any interpretation thereof held until the discussion.

Response: As suggested, we moved the interpretive statements about the LIWC analyses to the Discussion section.

Comment: In future iterations of the study, consider adding “Other/Non-binary” to gender options

Response: Thank you for this suggestion. We will be sure to do so in our future work.

Comment: Consider collecting demographic data that includes intended career pathway (helping professions vs other) and past experiences with smoking that might bias a participant (eg, does the study participant has a family member who smokes, or is the participant a current or past smoker? These are variables that may influence perspective-taking.

Response: Thank you for the suggestions about future demographic information to include. We did actually measure a few of the suggested items, but forgot to include them in the Participant section. As indicated in our experimental materials available on OSF, we collected the following three items about smoking: 1) Do you smoke cigarettes? (y/n); 2) At any point in your childhood, did one or both of your parents smoke cigarettes? (y/n/don’t know); 3) At any point in your childhood did your mother smoke? (y/n/don’t know). All of the demographic items are now summarized in the respective participant sections for both studies.

Comment: I did not see any discussion of limitations to the study. This should be added to the Discussion section. Examples: 1) Limiting participants to females (study 1) significantly limits generalizability of results, as females have been shown in numerous studies to statistically significantly test at higher empathy levels than males. 2)Limiting the study to undergraduate students also limits generalizability to health professionals.

Response: We added a discussion about the strengths and limitations of the work on pages 23-24.

Thank you for the opportunity to submit a revision of the manuscript. We appreciate the helpful comments, and we believe they have led to an improved version of the manuscript. We look forward to hearing your decision.

Sincerely,

Victoria A. Shaffer, PhD

Department of Psychological Sciences

University of Missouri

208 McAlester Hall

Columbia, MO 65211

Email: [shafferv@missouri.edu](mailto:shafferv@missouri.edu)
